# Drug-induced liver injury in COVID-19 treatment: Incidence, mechanisms and clinical management

**DOI:** 10.3389/fphar.2022.1019487

**Published:** 2022-11-28

**Authors:** Xichuan Li, Wanting Wang, Suying Yan, Weipeng Zhao, Hui Xiong, Cuiping Bao, Jinqian Chen, Yuan Yue, Yanjun Su, Chunze Zhang

**Affiliations:** ^1^ Tianjin Key Laboratory of Animal and Plant Resistance, College of Life Sciences, Tianjin Normal University, Tianjin, China; ^2^ Department of Colorectal Surgery, Tianjin Institute of Coloproctology, The Institute of Translational Medicine, Tianjin Union Medical Center of Nankai University, Tianjin, China; ^3^ Tianjin University of Traditional Chinese Medicine, Tianjin, China; ^4^ Department of Breast Cancer, Key Laboratory of Cancer Prevention and Therapy, Tianjin Medical University Cancer Institute and Hospital, National Clinical Research Center for Cancer, Tianjin, China; ^5^ School of Pharmaceutical Science and Technology, Tianjin University, Tianjin, China; ^6^ Departments of Radiology, Tianjin Union Medical Center, Tianjin, China; ^7^ Departments of Pharmacy, NHC Key Laboratory of Hormones and Development, Tianjin Key Laboratory of Metabolic Diseases, Tianjin Medical University Chu Hsien-I Memorial Hospital, Tianjin, China; ^8^ Department of Lung Cancer, Key Laboratory of Cancer Prevention and Therapy, Tianjin Lung Cancer Center, Tianjin Medical University Cancer Institute and Hospital, National Clinical Research Center for Cancer, Tianjin, China

**Keywords:** COVID-19, drug-induced liver injury, incidence, mechanisms, clinical management

## Abstract

The COVID-19 outbreak triggered a serious and potentially lethal pandemic, resulting in massive health and economic losses worldwide. The most common clinical manifestations of COVID-19 patients are pneumonia and acute respiratory distress syndrome, with a variety of complications. Multiple organ failure and damage, ultimately leading to patient death, are possible as a result of medication combinations, and this is exemplified by DILI. We hope to summarize DILI caused by the antiviral drugs favipiravir, remdesivir, lopinavir/ritonavir, and hydroxychloroquine in COVID-19 patients in this review. The incidence of liver injury in the treatment of COVID-19 patients was searched on PubMed to investigate DILI cases. The cumulative prevalence of acute liver injury was 23.7% (16.1%–33.1%). We discuss the frequency of these events, potential mechanisms, and new insights into surveillance strategies. Furthermore, we also describe medication recommendations aimed at preserving DILI caused by treatment in COVID-19 patients.

## 1 Introduction

By July 2022, the outbreak of Corona Virus Disease 2019 (COVID-19) has caused nearly 580 million confirmed diagnoses and over 6.4 million deaths. The most common early clinical symptoms of COVID-19 infection are fever, cough, myalgia, and fatigue. Approximately 15% of patients progress to an advanced stage of respiratory distress and eventually develop acute respiratory distress syndrome or multi-organ failure (Dong et al., 2020). Due of the scarcity of specific drugs at the onset of the COVID-19 epidemic, repurposed drugs were commonly utilized and mainly divided into antiviral and adjuvant drugs. Antiviral drugs included mainly remdesivir, hydroxychloroquine, lopinavir-ritonavir, azithromycin, oseltamivir, umifenovir, favipiravir, chloroquine, ribavirin, *etc.*; adjunctive drugs included antithrombotics, corticosteroids, antibiotics, metformin, vitamin supplements (C and D), antihypertensives, H2 receptor antagonists, and interleukin inhibitors. There are significant regional and temporal differences in the use of these medications. For example, the use of hydroxychloroquine is 85% in Spain, but less than 2% in China. Lopinavir-ritonavir was only used at the start of the pandemic in Korea and Spain, with a decreasing trend over time. Remdesivir shows a small upward trend from June 2020 (Prats-Uribe et al., 2021). According to clinical guidelines from various countries, antiviral drugs (such as favipiravir, remdesivir, lopinavir/ritonavir and hydroxychloroquine) are the most commonly used for COVID-19 treatment. Patients with comorbidities must also be treated for the underlying disease, and sedatives, anti-inflammatory, antipyretic, and analgesic drugs are used in critical patients. Multiple organ failure and damage, ultimately leading to patient death, are possible as a result of medication combinations, and this is exemplified by drug-induced liver injury (DILI) (Aithal et al., 2011).

Drug-induced hepatocellular injury is identified internationally by alanine aminotransferase (ALT) levels equal to or exceeding 5× the upper limit of normal (ULN) appearing within 3 months of drug initiation after alternative causes are excluded (Lee and Senior, 2005). When the suspect drug is removed, ALT usually drops by 50% or more. With drug re-administration, a positive rechallenge has recently been defined by an ALT level of 3–5× ULN or greater (Lammert et al., 2010). DILI is further affirmed by excluding other causes (e.g., viral hepatitis, biliary obstruction, alcoholic hepatitis, or hypotension), reports of suspect drug hepatotoxicity, and liver injury recurrence upon rechallenge (or re-administration) of the suspect drug, which has traditionally been strongly discouraged (Benichou et al., 1993; Danan and Benichou, 1993). As it is difficult to attribute the liver injury to drugs based on clinical indicators alone, a causality assessment scale is required for a definite diagnosis of DILI. The Council for International Organizations of Medical Sciences/Roussel Uclaf Causality Assessment Method (CIOMS/RUCAM) was the first attempt to standardize the concept of liver injury and is presently the most reliable and commonly used scale. It uses fractions to depict the probability of DILI: definite or highly probable (score>8), probable (score 6–8), possible (score 3–5), unlikely (score 1–2), excluded (score = 0). According to the biochemical mode of injury, drug-induced liver injury is characterized as hepatocellular, cholestatic, or mixed, and as intrinsic DILI and idiosyncratic DILI according to the mechanism of injury. The most frequent cause of intrinsic DILI is acetaminophen toxicity (Fisher et al., 2015). Diverse hypotheses exist about the underlying processes of idiosyncratic DILI, such as drug metabolism, inflammation, mitochondrial dysfunction, oxidative stress, and endoplasmic reticulum stress (Yuan & Kaplowitz, 2013). The treatment of DILI mainly relies on early diagnosis and withdrawal of suspected drugs. Corticosteroids and ursodeoxycholic acid may be used as a supplementary therapy. Plasma replacement and liver transplantation may be the sole remaining choices at the stage of liver failure.

It is undeniable that COVID-19 can also induce liver injury. Severe acute respiratory syndrome coronavirus 2 (SARS-CoV-2) may directly bind to angiotensin-2 converting enzyme (ACE2) positive cholangiocytes (Hoffmann et al., 2020). Moreover, activation of the immune system and cytokine storm may contribute to an immune-mediated process of hepatic injury in COVID-19 (Alqahtani & Schattenberg, 2020). Endotheliopathy, altered platelet function, inflammation, and their synergistic effects may lead to liver injury in patients (McConnell et al., 2021). However, this liver injury is not drug-induced and is therefore outside the subject of this article.

Due to a large number of asymptomatic cases and the small number of cases in which rigorous clinical testing for DILI is performed during COVID-19 treatment, it is difficult to determine the absolute incidence of DILI. Therefore, the above technical definition of DILI does not apply to this review. In this article, we take into account data on abnormal liver function tests linked to drugs. We retrieved previously published articles from PubMed in order to analyze the incidence of drug-induced abnormal liver function in patients with COVID-19, in conjunction with drug pharmacokinetics to speculate on possible mechanisms and to provide reasonable clinical management recommendations.

## 2 High incidence of abnormal liver function during the antiviral treatment of COVID-19 patients

There is a high incidence of liver injury in patients with COVID-19. According to a systematic evaluation performed in September 2020, the cumulative prevalence of acute liver injury among COVID-19 patients was 23.7% (16.1%–33.1%) (Kumar et al., 2020). In another systematic review and meta-analysis, the frequency of liver injury in COVID-19 patients was 19% (1%–53%) (Mao et al., 2020). Several antiviral drugs in the COVID-19 regimen are repurposed drugs that have previously been reported to cause DILI. Lopinavir/ritonavir is primarily used as an HIV therapy medicine in the clinic. The incidence of hepatotoxicity after antiretroviral therapy (ART) in hepatitis B and C patients with HIV was found to be 40.1/100 py in the lopinavir/ritonavir group (Su et al., 2018). In addition, hydroxychloroquine has been reported to cause severe DILI in the treatment of porphyrias (Sunkara et al., 2018). Therefore, we summarized the incidence of abnormal liver function during the antiviral treatment of COVID-19 patients in Table 1.

**TABLE 1:** Incidence of abnormal liver function and medication regimen during the antiviral treatment of COVID-19 patients.

Antiviral drugs	References	Dose	Duration	Frequency	Incidence	Research method	Location
Favipiravir	Tabarsi et al.	1600 mg	1d	Twice daily	9% of abnormal liver function	RCT	Iranian
		600 mg	5d	Twice daily			
	Chen et al.	1600 mg	1d	Twice daily	8.62% of abnormal liver function	RCT	China
		600 mg	6d	Twice daily			
	Udwadia et al.	1800mg	1d	Twice daily	6.8% of abnormal liver function	RCT	India
		800mg	13d	Twice daily			
	Lou et al.	1600mg/2200mg	first dose	Not mentioned	11% of elevated AST 44% of elevated ALT 11% of elevated total bilirubin	RCT	China
		600 mg	≤14d	Three times daily			
	Ergür et al.	1600 mg	5-10d	Twice daily	7.28% of elevated transaminases	Retrospective	Turkey
		600 mg		Twice daily			
Remdesivir	Grein et al.	200mg	1d	Not mentioned	23% of increased liver enzymes	Retrospective	USA, Japan, Italy, France
		100mg	9d	Not mentioned			
	Antinori et al.	200mg	1d	Not mentioned	42.8% of elevated transaminases	Prospective	Italy
		100mg	9d	Not mentioned			
	Ader et al.	200mg	1d	Once daily	3% of elevated transaminases	RCT	48 sites in Europe
		100mg	9d	Once daily			
	Goldman et al.	200mg	1d	Once daily	6.5% of elevated ALT	RCT	USA, Italy, Spain, Germany, Hong Kong, Singapore, Korea, Taiwan
		100mg	9d/4d	Once daily	5.8% of elevated AST		
	Beigel et al.	200mg	1d	Not mentioned	3.4% of elevated AST	RCT	USA, Denmark, UK, Greece, Germany, Korea, Mexico, Spain, Japan, Singapore
		100mg	9d	Not mentioned	1.7% of elevated total bilirubin		
	Wang et al.	200mg	1d	Once daily	5% of elevated AST	RCT	China
		100mg	9d	Once daily	10% of elevated total bilirubin		
	Kanai et al.	100mg	1d	Not mentioned	46.2% of abnormal liver function	Retrospective	Japan
		200mg	9d	Not mentioned			
	van Laar et al.	Not mentioned	Not mentioned	Not mentioned	43% of elevated ALT 45% of elevated AST	Retrospective	The Netherlands
	Leegwater et al.	Not mentioned	5d	Not mentioned	elevated ALT (1305 U/L) elevated AST (1461 U/L)	Case	The Netherlands
	McCoy et al.	200mg	1d	Not mentioned	elevated liver enzymes	Case	USA
		100mg	9d	Not mentioned			
	Kaur et al.	5mg/kg/d	1 dose	Not mentioned	elevated ALT (832 U/L) elevated AST (1121 U/L)	Case	India
Lopinavir/Ritonavir	Fan et al.	Not mentioned	Not mentioned	Not mentioned	63.4% of abnormal liver function	Retrospective	China
	Zhu et al.	400mg/100mg	7d	Twice daily	8.8% of elevated ALT(<125U/L)	Retrospective	China
	Cao et al.	400mg/100mg	14d	Twice daily	2.1% of elevated AST;1.1% of elevated ALT 3.2% of elevated total bilirubin	RCT	China
Hydroxychloroquine	Cavalcanti et al.	400mg	7d	Twice daily	8.5% of elevated ALT/AST 2.5% of elevated total bilirubin	RCT	Brazil
	Ader et al.	400mg	1d	Twice daily	4% of elevated transaminases	RCT	France
		400mg	9d	Once daily			
	Satlin et al.	600mg	1d	Twice daily	10.7% of elevated AST;8.1% of elevated ALT ;1.6% of elevated ALP	Retrospective	USA
		400mg	4d	Once daily	3.3% of elevated total bilirubin all of which were grade 3 or 4 adverse effects		
	Falcão et al.	400mg	1d	Twice daily	AST from 46 to 469 U/L ALT from 33 to 357 U/L	Case	Brazil
	Hillaker et al.	400mg	1d	Twice daily	elevated ALT, AST	Case	USA
		200mg	4d	Twice daily

### 2.1 Favipiravir

Favipiravir is a purine analogue and RNA-dependent polymerase inhibitor that has been shown to be effective against a variety of RNA viruses, including the Ebola virus. In two prospective, randomized controlled clinical studies that included patients with moderate to severe COVID-19 but excluded those with severe liver disease, similar incidences of abnormal liver function were found to be 9% and 8.62% in the favipiravir group, respectively (Chen et al., 2021; Tabarsi et al., 2021). Another clinical trial that included only patients with mild to moderate (including asymptomatic) COVID-19 obtained an incidence of 6.8% (Udwadia et al., 2021). Interestingly, in a small exploratory randomized controlled trial comparing baloxavir and favipiravir, one case (11%) of elevated aspartate aminotransferase (AST), four cases (44%) of elevated ALT, and one case (11%) of elevated total bilirubin were found in the favipiravir group, a significantly higher proportion compared to other trials. We believe this is related to the small number of patients included in the favipiravir group (9 cases), as patients with abnormal baseline liver function parameters were not excluded (Lou et al., 2021). An additional retrospective study included 357 favipiravir-treated patients, with 26 (7.28%) having elevated transaminases. The participants were divided into groups based on the existence or absence of side effects, and it was discovered that there was a positive correlation between elevated body mass index (BMI), baseline transaminases, and ferritin levels with the occurrence of side effects (Ergür et al., 2022).

### 2.2 Remdesivir

Remdesivir is a nucleotide monophosphate analogue prodrug that inhibits viral RNA-dependent RNA polymerase. It has antiviral activity against a broad spectrum of human coronaviruses, including SARS-CoV-2, in cell cultures and mouse models. The Food and Drug Administration (FDA) and European Medicines Agency (EMA) recommend remdesivir for the treatment of COVID-19 infection based on data from three randomized controlled trials (Eastman et al., 2020).

Remdesivir was discovered to be hepatotoxic during clinical trials. An evaluation of the adverse drug reactions associated with remdesivir in the VigiBase database revealed that elevated liver enzymes accounted for 32.1% of the cases (Charan et al., 2021). High incidences of increased liver enzymes were also found in several clinical trials including mechanically ventilated patients, with rates of 23% and 42.8%, respectively (Antinori et al., 2020; Grein et al., 2020). However, a similar trial with a rate of 3% was also conducted (Ader et al., 2022), and it has been argued that the large difference in rates is due to the fact that the study explicitly excluded people who were taking other antivirals at the same time. In comparison, the incidence of liver enzyme elevations appeared to be lower in patients who did not receive mechanical ventilation, with 26 (6.5%) ALT and 23 (5.8%) AST elevations, respectively, as shown in another clinical trial (Goldman et al., 2020). Similar results were seen in two trials comparing the effects of remdesivir to placebo control, with ASTs raised by 5% and 3.4%, but with total bilirubin raised by 10% and 1.7%, respectively (Wang Q. et al., 2020; Beigel et al., 2020). According to one study, 46.2% of COVID-19 patients aged 80 and older had liver dysfunction, which was significantly higher than the frequency for people under the age of 80 (Kanai et al., 2021). In a retrospective study, 43% and 45% of patients with normal baseline ALT and AST showed elevations after remdesivir treatment, respectively (van Laar et al., 2021). As the study failed to control for variables, there is no assurance that remdesivir was the sole independent factor causing hepatotoxicity in these patients.

Several case reports describe the correlation between remdesivir and liver injury in more detail. A 64-year-old male patient presented with a sharp increase in ALT (1305 U/L) and AST (1461 U/L) after 5 days of remdesivir. After discontinuing the drug immediately, ALT and AST levels decreased rapidly and eventually returned to normal levels. After analyzing the time points of changes in ALT/AST levels, the author ruled out liver injury caused by COVID-19 and amiodarone. He concluded that the patient’s elevated liver enzyme levels were most likely caused by remdesivir (Leegwater et al., 2021). Five pregnant women infected with COVID-19 were treated with remdesivir, and four of them developed elevated liver enzymes, prompting one of them to discontinue the medication (McCoy et al., 2020). Another case report describes a newborn with COVID who had a significant increase in ALT/AST (ALT 832 U/L; AST 1121 U/L) after receiving the first dose of remdesivir, which was then discontinued. After 10 days, liver enzyme levels returned to normal. The timing correlation between changes in liver enzyme levels and the use of remdesivir in this case suggested that drug-induced liver injury was more likely (Kaur et al., 2022).

### 2.3 Lopinavir/ritonavir

Lopinavir/ritonavir is a fixed-dose combination antiretroviral drug that is widely used for HIV/AIDS prevention and treatment. At the beginning of the pandemic, it emerged as a potential candidate for the treatment of COVID-19 (Osborne et al., 2020). Using data from the FDA Adverse Event Reporting System, the incidence of DILI in COVID-19 patients treated with lopinavir/ritonavir was analyzed. The results showed that 313 (37%) of 845 adverse reactions were DILI (Tang et al., 2021).

According to a retrospective study, 63.4% of 41 COVID-19 patients treated with lopinavir/ritonavir had abnormal liver function tests. The study divided 93 patients with normal baseline liver function into two groups based on the presence or absence of abnormalities after therapy. According to the findings, 57.8% of the abnormal group received lopinavir/ritonavir, which was significantly higher than the normal group (*p* < 0.01) (Fan et al., 2020). Furthermore, lopinavir/ritonavir was associated with an increased risk of liver injury, defined as ALT and or AST >3×ULN, ALP, GGT and TBIL (alkaline phosphatase,γ-glutamyltransferase and total bilirubin) > 2×ULN (OR from 4.44 to 5.03, *p* < 0.01) (Cai et al., 2020). The incidence in another study was 8.8%, which is significantly lower than the incidence in the preceding studies. The effect of their included population’s younger age (median age = 40 years) and smaller sample size cannot be ruled out (Zhu Z. et al., 2020). To reduce confounding factors, a prospective study included patients with severe COVID-19 and excluded those with severe liver disease and AIDS, which may explain their low percentage of elevated AST, ALT, and total bilirubin (1.1%, 2.1%, and 3.2%, respectively) (Cao et al., 2020).

### 2.4 Hydroxychloroquine

Hydroxychloroquine is a 4-aminoquinoline compound, a derivative of quinine, previously used for the prevention and treatment of malaria and some rheumatic diseases such as systemic lupus erythematosus (Colson et al., 2020). During *in vitro* testing, hydroxychloroquine effectively inhibited SARS-Cov-2 infection, possibly *via* two main mechanisms (Liu et al., 2020), one of which was the inhibition of SARS-Cov-2 entry into human cells and prevention of its replication, and the other of which was the prevention of fulminant COVID-19, including cytokine release syndrome (Al-Bari, 2017; Zhou D. et al., 2020; Zhou P. et al., 2020). In clinical studies with 665 participants, 199 were given hydroxychloroquine alone; 17 (8.5%) had elevated ALT/AST levels, while 5 (2.5%) had elevated bilirubin levels (Cavalcanti et al., 2020). Two other prospective trials yielded similar incidences of liver enzyme abnormalities (4% and 6.7%, respectively) (Chen J. et al., 2020; Ader et al., 2021). The results of a retrospective cohort study showed that among COVID-19 patients who had received one dose of hydroxychloroquine, 13 (10.7%) had elevated AST, 10 (8.1%) had elevated ALT, 2 (1.6%) had elevated ALP, and 4 (3.3%) had elevated total bilirubin, all of which were grade 3 or four adverse effects (Satlin et al., 2020).

Two case reports also describe liver dysfunction caused by hydroxychloroquine. The first case was a 29-year-old female patient who had just delivered at full term and had an approximately 10-fold increase in transaminase levels after two doses of hydroxychloroquine (AST from 46 to 469 U/L; ALT from 33 to 357 U/L), after which the drug was subsequently discontinued. Transaminase levels returned to near-normal values after 5 days (Falcão et al., 2020). In another case of a 40-year-old male, transaminases were elevated after 5 days of hydroxychloroquine; then stop taking the medication. Unfortunately, the report did not go into detail about the elevated transaminase values (Hillaker et al., 2020).

## 3 Possible mechanisms of liver injury caused by antiviral agents

The entire process of drug uptake into the liver, metabolism, and final excretion is controlled by the large families of proteins (Yuan & Kaplowitz, 2013). Drugs are passively taken up into hepatocytes or by a series of transporters located in the basolateral plasma membrane, including members of the solute carriers (SLCs), organic anion-transporting polypeptides (OATPs), organic anion-transporter (OAT) family, and organic cation transporters (OCTs) (Burckhardt & Burckhardt, 2011; Hagenbuch & Stieger, 2013). Drugs are metabolized by phase I and phase II enzymatic reactions after ingestion. Phase I metabolism is primarily concerned with the oxidation and reduction of drugs by cytochrome P450 (CYP450) to generate reactive metabolites, whereas phase II metabolism is concerned with the binding of drugs or phase I metabolites to endogenous molecules (Yuan & Kaplowitz, 2013). The ATP Binding Cassette (ABC) transporters then mediate the efflux of the drug and its metabolites from the hepatocytes into the bile or back into the blood sinusoids for subsequent renal excretion (Andrade et al., 2019). Hepatocyte exposure to increased cellular stress is assumed to be the initial step in DILI development. Initial cell damage is induced by drugs and/or their reactive metabolites *via* covalent binding or direct damage to mitochondria, which leads to oxidative stress and the activation of stress-sensing signaling pathways, impairment of the mitochondrial function, and endoplasmic reticulum (ER) stress. The production of reactive metabolites during drug metabolism results in a major rise in mitochondrial oxidative stress, with the aggravation of reactive oxygen species (ROS) further damaging cells and tissues. The production of reactive oxygen species (ROS) from injured hepatocytes increases overall oxidative stress, and the release of damage-associated molecular patterns (DAMP) activates innate immune responses, resulting in the activation of apoptotic and necrotic pathways (Garcia-Cortes et al., 2020; Villanueva-Paz et al., 2021).

**FIGURE 1:**
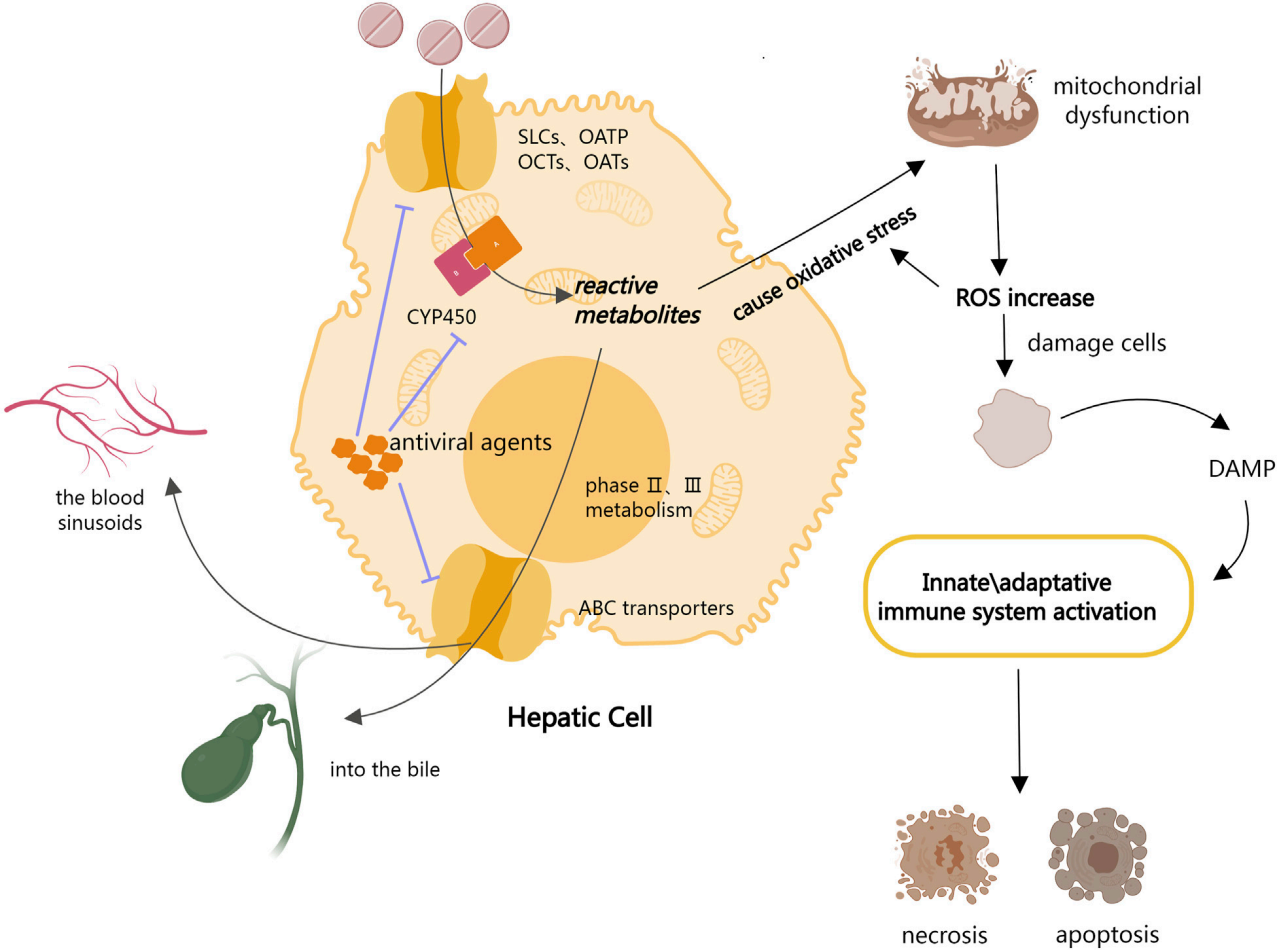
Metabolism of antiviral drugs in hepatocytes leads to DILI.

According to the drug metabolism process described above, antiviral drug entry into the liver inhibits the activity of CYP enzymes and transporter proteins, which may affect the redox and excretion of drugs metabolized *via* these two pathways (Weemhoff et al., 2003; Ambrus et al., 2021). This may lead to a homeostasis imbalance, which in turn causes DILI (Figure 1). Moreover, according to the National Institutes of Health Guidelines 2021 for the Treatment of Novel Coronavirus Pneumonia, antiviral agents are frequently used to treat COVID-19. The cumulative prevalence of acute liver injury among COVID-19 patients was 23.7% (16.1%–33.1%) (Kumar et al., 2020). Therefore, we hypothesize that antiviral medications may cause DILI in COVID-19 patients by inhibiting CYP enzymes and liver transport proteins.

### 3.1 Inhibition of CYP by antiviral agents

CYP450 is a class of hemoglobin-coupled monooxygenases found primarily in the endoplasmic reticulum of the liver and other tissues, and it functions with the coenzyme nicotinamide adenine dinucleotide phosphate (NADPH) and molecular oxygen. P450 enzymes are the primary enzyme system responsible for drug metabolism and are primarily involved in oxidative reactions during drug biotransformation (Takahashi et al., 2020). Multiple CYP450 isoforms are present in liver microsomes, with CYP3A4 accounting for 30% of total CYP in the liver. Inhibiting CYP3A4 affects drug metabolism in the liver, causing liver injury. Previous research has shown that remdesivir and lopinavir/ritonavir are CYP3A4 inhibitors, while remdesivir also inhibits CYP1A2, CYP2C9, CYP2C19, and CYP2D6; lopinavir/ritonavir and hydroxychloroquine both inhibit CYP2D6 (Kim et al., 2003; Yang, 2020). Furthermore, favipiravir has also been reported as an inhibitor of CYP2C8 (Deb and Arrighi, 2021).

Despite the fact that many studies have found that the aforementioned drugs inhibit CYP, the strength of their inhibitory effect on CYP remains inconclusive. Lopinavir’s inhibition of six CYP enzymes was systematically assessed using a human liver microsomal model, and lopinavir was found to inhibit all five CYP enzymes weakly except CYP3A4, which was moderately inhibited (Weemhoff et al., 2003). Lopinavir/ritonavir is also a weak inhibitor of CYP2C9 (Lim et al., 2004) (Table 2).

**TABLE 2:** Relationship between antiviral agents and CYP or transporters.

Antiviral agents	Inhibitors of CYP	Inhibitors of transporters	Substrates of CYP	Substrates of transporters
Favipiravir	CYP2C8	-	-	-
Remdesivir	CYP3A4, CYP1A2, CYP2C9, CYP2C19, CYP2D6	OATP1B1, OATP1B3, OATP2B1, OCT1, ABCC4	CYP3A4, CYP2C8, CYP2D6	-
Lopinavir/ritonavir	CYP3A4, CYP2D6, CYP2C9	ABCC2, ABCB1, ABCB3, ABCB11, ABCG2, OATP1B1, OATP1B3	CYP3A4	OATP1A2, OATP1B1, OATP1B3, ABCB1, ABCC2
Hydroxychloroquine	CYP2D6	ABCB1	CYP3A4, CYP2D6	-

### 3.2 Inhibition of transporters by antiviral agents

Hepatic transporters play an important role in the clearance of endogenous and exogenous substances, and the main common transporter proteins are the following:

The ABCC2/MRP2 transporter (ATP Binding Cassette Subfamily C member 2/Multidrug Resistance-Associated Protein 2) mediates the entry of endogenous and exogenous substances into bile. It is a multi-specific transporter of amphiphilic compounds and a key transporter for bilirubin conjugates. OATP1B1 and OATP1B3 are organic anion uptake transport proteins in the liver that are involved in the uptake and elimination of various drugs and toxic compounds from the bloodstream. OCT1 is a highly expressed organic cation uptake transporter in the liver. It allows nutrients to enter cells and can mediate drug uptake in patients. The levels of OCT1 expression correlate with the responses toward many drugs. The interaction between antiviral agents and hepatic transporters may result in hepatotoxicity and adverse drug effects (Giacomini et al., 2010).

The liver drug uptake transporters OATP1B1, OATP1B3, OATP2B1 and OCT1 were found to have low remdesivir uptake ratios by cellular transfection, indicating that the transporters were not related to remdesivir hepatic uptake (Nies et al., 2021). However, remdesivir inhibits the transporters in a concentration-dependent manner and can function as an inhibitor. And remdesivir has been shown to inhibit at 10 M concentrations, which is close to the peak plasma concentration observed 30 min after receiving 200 mg remdesivir intravenously (Jorgensen et al., 2020). Another study also demonstrated that remdesivir inhibits OCT1 *in vitro*. According to this study, remdesivir inhibited MRP4 but not MRP2 or MPR3 (Ambrus et al., 2021).

Lopinavir was found to have the most significant inhibitory effect on ABCC2/MRP2 by observing bile accumulation of CDF (cumulative distribution function), a visualization fluorescent substrate, while ritonavir’s effect was not statistically significant (Holmstock et al., 2018). Some studies support lopinavir’s inhibitory effect on ABCC2/MRP2, while other studies suggest that ritonavir similarly inhibits ABCC2/MRP2 (Drewe et al., 1999; Huisman et al., 2002; Agarwal et al., 2007; Ye et al., 2010). Besides ABCC2/MRP2, lopinavir/ritonavir has been shown to inhibit other ABC transporter proteins, including ABCB1 (Perloff et al., 2001), ABCB3 (Ernest et al., 2005), ABCB11 (Pedersen et al., 2013), and ABCG2 (Drewe et al., 1999; Profit et al., 1999; Ernest et al., 2005; Gupta et al., 2004). Favipiravir had no significant effect on any of these transporters (Ambrus et al., 2021).

Hydroxychloroquine inhibited ABCB1/P-gp (P-glycoprotein) at concentrations exceeding 10 µM. Although hydroxychloroquine has a low interaction potential with transporters, it may increase the bioavailability of concurrently administered ABCB1/P-gp substrates (Weiss et al., 2020) (Table 2).

### 3.3 Interactions between different antiviral agents

Antiviral agents are metabolized as substrates of CYP and transporters in addition to acting as inhibitors of them. Remdesivir has been identified as a substrate for CYP3A4, CYP2C8, and CYP2D6 (Yang et al., 2020); hydroxychloroquine is metabolized by CYP3A4 and CYP2D6 (Deb and Arrighi, 2021); and lopinavir/ritonavir is also metabolized by CYP3A4 (Cao et al., 2020). Lopinavir was confirmed as a substrate for OATP1A2, OATP1B1, and OATP1B3 by examining the substrate specificity of antiretroviral drugs using oocyte cell lines and analyzing the association between SNP and lopinavir plasma concentrations using plasma samples (Hartkoorn et al., 2010). Furthermore, lopinavir is also a substrate for ABCB1 and ABCC2 (Woodahl et al., 2005) (Table 2). CYP and transporter proteins act as mediators of antiviral drug interactions in these cases. This could be one of the mechanisms of liver damage caused by the combination of antiviral drugs used to treat COVID-19 patients.

### 3.4 Interactions between antiviral agents and other COVID-19 clinical agents

#### 3.4.1 CYP-mediated drug-drug interactions

In the clinical of COVID-19, in addition to antiviral agents, patients may receive medications for sedation during ventilation and medications for comorbidities (e.g., heart disease, diabetes, hypertension, hyperlipidemia, *etc.*). These drugs are metabolized by CYP and may interact with CYP inhibitors to produce DILI.

Propofol, a sedative commonly used in patients with mechanical ventilation, is metabolized by CYP2B6 (Meyer and Maurer, 2011). Fentanyl, benzodiazepine drugs, and midazolam are also commonly used sedatives, all of which are metabolized by CYP3A4 (Meyer and Maurer, 2011; Doi et al., 2020). CYP3A4 inhibitors, such as lopinavir/ritonavir, may affect their pharmacokinetics and toxicity when combined. Epidemiological studies have shown that people with hypertension, diabetes, and hyperlipidemia are more likely to be infected with COVID-19 (Aitken and Morgan, 2007; Croyle, 2009), which means they are more likely to develop drug-drug interactions (DDI) with antiviral agents in the clinical setting and should be taken more seriously. Most dihydropyridine calcium channel blockers (e.g., amlodipine and nifedipine) (Sutton et al., 1997; Katoh et al., 2000), all non-dihydropyridine calcium channel blockers (e.g., verapamil and diltiazem) (Tateishi et al., 1992), and propranolol (Zhang et al., 1998) are metabolized by CYP3A4. Irbesartan and losartan are metabolized by CYP2C9 (McCrea et al., 1999). Cholesterol-lowering drugs, such as statins (except pravastatin and rosuvastatin) are metabolized by CYP3A4 (Neuvonen and Jalava, 1996; Cook et al., 2002). Glimepiride, glipizide, and glyburide are all anti-diabetic drugs that are metabolized by CYP2C9 (Suzuki et al., 2006). Because remdesivir inhibits both CYP2C9 and CYP3A4, extra attention must be paid to the multiple effects of drug-drug interactions when administering remdesivir to the aforementioned patients. It is recommended that blood levels and related indicators of DILI be monitored on a regular basis, or that alternative drugs that are not metabolized by CYP, such as angiotensin-converting enzyme (ACE) inhibitors (Hoyer et al., 1993), thiazide diuretics (Ellison, 2019) be considered.

#### 3.4.2 Liver transporters-mediated drug-drug interactions

In addition to drugs metabolized by CYP, some drugs are metabolized with the involvement of transporters. Transporter inhibitors can also affect such drugs, resulting in drug-drug interactions. Statins make up a large portion of the comorbid medications used in COVID-19 patients, and one of their side effects is hepatotoxicity. A previous study showed that breast cancer resistance protein (BCRP) and P-gp transported atorvastatin, fluvastatin, pitavastatin, and rosuvastatin; MRP2 transported fluvastatin; MRP3 transported atorvastatin, fluvastatin, pitavastatin, and pravastatin; and MRP4 transported fluvastatin and rosuvastatin (Deng et al., 2021). Lopinavir/ritonavir, as a potent MRP2 inhibitor, can affect the excretion of some of these drugs and may exacerbate their adverse effects. Clopidogrel carboxylate (CPC), the inactive metabolite of clopidogrel, was identified as a substrate of OAT1 through *in vitro* experiments. Additionally, metformin is identified as a substrate of OCT1 (Li et al., 2014). Several macrolides, including clarithromycin, roxithromycin, telithromycin, azithromycin, and erythromycin, have been identified as ABCB1/P-gp substrates (Munić et al., 2010). In the clinical use of COVID-19 patients, other drugs may be metabolized by liver transporters. When these drugs are combined with antiviral agents that inhibit transporters, the possibility of liver injury cannot be ruled out, so caution is advised.

## 4 Clinical management

### 4.1 Indicators for monitoring

In COVID-19 hospitalized patients, the incidence of abnormal liver function tests ranges from 10.5 to 69% (Mao et al., 2020). Most studies show that abnormal liver function tests are primarily caused by AST and ALT elevations, with AST elevations being more common than ALT elevations (Cai et al., 2020). Elevations in GGT and total bilirubin are less common than AST and ALT elevations (Lala et al., 2022). According to previous research, the proportion of COVID-19 patients with elevated ALT was 9.6–37.6% (Guan et al., 2020; Bloom et al., 2021), elevated AST was 14.8–36% (Zhang Y. et al., 2020; Xie et al., 2020), abnormal GGT was 13.0–24.4% (Chen N. et al., 2020; Hao et al., 2020), and abnormal total bilirubin was 5.1–18% (Zhang C. et al., 2020; Zhu J. et al., 2020; Huang et al., 2020).

The majority of patients with abnormal liver function have mildly elevated AST/ALT (1–2 times the ULN), with only a minority (<4%) having levels greater than 2 times the ULN. GGT is significantly higher than other indicators and may exceed three times the ULN (Cai et al., 2020). By the time of discharge, the majority of mild COVID-19 patients had normalized their indicators, whereas severe patients were more likely to have not returned to normal levels (Wang Y. et al., 2020). Therefore, all COVID-19 patients should be tested regularly for these biochemical parameters, and re-testing is recommended in severe patients after discharge until liver function levels return to normal. Furthermore, uric acid values should also be monitored with favipiravir, and extra attention should be paid to neutrophils and platelets with remdesivir and lopinavir/ritonavir (Marc et al., 2021). A liver biopsy is recommended for patients with one of the following three conditions:(a) Persistent elevation of hepatic biochemical parameters or signs of deterioration in liver function after discontinuation of the suspected drug; (b) cases of DILI where continued use or re-exposure to the implicated agent is contemplated; (c) liver biochemistry abnormalities persist beyond 180 d, especially if associated with symptoms (e.g., itching) or signs (e.g., jaundice and hepatomegaly) (Chalasani et al., 2021).

### 4.2 Medication recommendations

#### 4.2.1 General medication recommendations

The dosage and duration of antiviral agents may adversely affect liver metabolism and result in liver injury (Kumar et al., 2021). To minimize liver damage, regular monitoring of the relevant indicators, flexible-dose adjustment, and timely discontinuation of the drug are required. Remdesivir is not recommended for patients with baseline ALT ≥5 x ULN, and should be stopped if any of the following conditions occur during dosing: (I) ALT ≥5 x ULN; (II) ALT elevation accompanied by signs or symptoms of liver inflammation; or (III) ALT elevation accompanied by elevated conjugated bilirubin, ALP or international normalized ratio (INR). When ALT<5×ULN, the drug can be restarted again (Lamb, 2020; Malin et al., 2020). Since hydroxychloroquine accumulates in the liver, it is critical to continuously monitor the patient’s liver function throughout clinical treatment and to be cautious when it is combined with other hepatotoxic drugs (Piszczatoski and Powell, 2020). Lopinavir/ritonavir is contraindicated in patients with severe liver injury because it has not been studied in this population (Stower, 2020). While no dose reduction is required for patients with mild to moderate liver damage, liver function tests (LFTs) must be closely monitored (Marra et al., 2021) (Table 3).

**TABLE 3:** Medication recommendations of antiviral drugs.

Drugs	Medication recommendations		
	GeneralRecommendations	For Patients with Hepatic Dysfunction	For Patients withComorbidities
**Remdesivir**	(i) not recommended for patients with baseline ALT ≥5 x ULN	(i)be used without dose adjustment in patients with hepatic dysfunction if there are no contraindications and the clinical benefits outweigh the risks	careful dosage consideration
	(ii)be stopped if any of the following conditions occur during dosing: (I) ALT ≥5 x ULN; (II) ALT elevation accompanied by signs or symptoms of liver inflammation; or (III) ALT elevation accompanied by elevated conjugated bilirubin, ALP or INR	(ii)not recommended if the patient’s baseline ALT is > 5 x ULN	close monitoring
	(iii)ALT<5×ULN, the drug can be restarted again
**Lopinavir/Ritonavir**	(i)contraindicated in patients with severe liver injury	(i)no dosage reduction is required, but LFTs should be closely monitored	careful dosage consideration
	(ii)no dose reduction is required for patients with mild to moderate liver damage, LFTs must be closely monitored	(ii)not recommended for use in patients with severe hepatic dysfunction on COVID-19	close monitoring
**Hydroxychloroquine**	continuously monitor and to be cautious	(i)50% loading dose as a maintenance dose and a maximum dosage of no more than 400 mg per day for patients with severe hepatic dysfunction (CPT C)	careful dosage consideration
		(ii)be reduced in patients with mild to moderate hepatic dysfunction if there are other risks of toxicity	close monitoring
**Favipiravir**	-	(i)CPT A and CPT B patients receive the same dose as the healthy population
		(ii)CPT C patients receive a lower dose and a shorter dosing schedule	-

#### 4.2.2 Medication recommendations for high-risk groups

##### 4.2.2.1 For patients with hepatic dysfunction

###### 4.2.2.1.1 Favipiravir

The dosage and duration of favipiravir should be modified based on the Child-Pugh Test (CPT). We recommend that CPT A and CPT B patients receive the same dose as the healthy population, while CPT C patients receive a lower dose and a shorter dosing schedule, based on prescribing information (Jafari et al., 2020) and the clinical trial of Preston R.

###### 4.2.2.1.2 Remdesivir

Since remdesivir is primarily metabolized by the kidney, it can be used without dose adjustment in patients with hepatic dysfunction if there are no contraindications and the clinical benefits outweigh the risks (Marra et al., 2021; Sahakijpijarn et al., 2021). The main adverse effect of remdesivir is elevated hepatic transaminases, and this drug is not recommended if the patient’s baseline ALT is > 5 x ULN (Sahakijpijarn et al., 2021).

###### 4.2.2.1.3 Lopinavir/ritonavir

Although the AUC (Area Under Curve) of lopinavir was 30% higher in patients with mild to moderate hepatic dysfunction than in patients with normal liver function, no clear correlation with clinical treatment was observed (Peng et al., 2006). Consequently, no dosage reduction is required, but LFTs should be closely monitored (Li et al., 2020). Lopinavir/Ritonavir may aggravate liver dysfunction based on the fact that it can cause elevated liver enzymes and bilirubin (Li et al., 2020). Furthermore, no studies in patients with severe hepatic dysfunction have been conducted, so it is not recommended for use in patients with severe hepatic dysfunction on COVID-19.

###### 4.2.2.1.4 Hydroxychloroquine

As hydroxychloroquine accumulates in the liver, it should be used with caution in patients with COVID-19, despite the limited duration of dosing (Ferron et al., 2021). For patients with severe hepatic dysfunction (CPT C), a conservative regimen is to use a 50% loading dose as a maintenance dose and a maximum dosage of no more than 400 mg per day. The dosage should also be reduced in patients with mild to moderate hepatic dysfunction if there are other risks of toxicity. Moreover, baseline monitoring of liver function and ongoing monitoring of toxicity remain essential (Marra et al., 2021).

Other recommendations include that antiviral therapy for HBV should be continued, but antiviral therapy for HCV patients may need to be delayed. Non-emergency patients may postpone liver ultrasounds or biopsies. Strict indications for treatment should be followed when starting immunosuppressive drugs in patients with liver disease such as autoimmune hepatitis or graft rejection. Immunosuppression should be continued in patients with AIH or transplantation (Sucher et al., 2019; Li et al., 2020).

##### 4.2.2.2 For patients with comorbidities

Common comorbidities among COVID-19 patients include coronary artery disease, hypertension, diabetes, and hyperlipidemia, and this population requires an additional targeted drug. The mechanisms by which these drugs interact with antiviral drugs have previously been described. Furthermore, antiviral medications may exacerbate comorbidities. Here, we recommend adjusting the dosage and dosing intervals or switching to a different drug with no interactions.

The clinical medication in elderly COVID-19 patients should be approached with greater caution, due to their diminished physiological functions, decreased liver and kidney metabolic functions, and the more possibility of concomitant coronary heart disease, diabetes mellitus, hypertension, hyperlipidemia, and other underlying diseases. The pharmacokinetic studies of remdesivir and lopinavir/ritonavir did not include elderly patients (age >65), so careful dosage consideration and close monitoring of relevant indicators are advised when administering these antiviral agents (Zanon et al., 2020; Sahakijpijarn et al., 2021). Hydroxychloroquine has few adverse reactions and can be used in COVID-19 elderly patients, who should be focused on monitoring adverse reactions of cardiac, ocular, and renal.

## 5 Conclusion

The cumulative incidence of liver injury among COVID-19 patients was alarmingly high at 23.7% (16.1%–33.1%). The incidence of liver function abnormalities linked to favipiravir ranged from 6.8% to 44%, remdesivir from 1.7% to 46.2%, lopinavir-ritonavir from 1.1% to 63.4%, and hydroxychloroquine from 1.6% to 10.7%, according to our review of the data. Antiviral medicines have an inhibiting effect on CYP enzymes and liver transport proteins, which may account for these elevated incidences. Antiviral drugs inhibit CYP enzymes and hepatic transporter proteins, resulting in a buildup of reactive chemicals that initiate a cascade of biochemical stress reactions, finally resulting in hepatocyte necrosis and apoptosis.

The COVID-19 epidemic has been ongoing for 3 years, and clinical guidelines from various countries are continually updated. According to the most recent WHO guidelines, hydroxychloroquine and lopinavir/ritonavir are not recommended, and remdesivir is only recommended for conditional use in serious patients. Two new antiviral drugs are recommended: conditional recommendation against the use of nirmatrelvir-ritonavir (Paxlovid) in patients with non-severe illness at low risk of hospitalization; conditional recommendation for the use of molnupiravir in patients with non-severe COVID-19, at highest risk of hospitalization. A meta-analysis study demonstrated that molnupiravir and Paxlovid were effective in reducing mortality and hospitalization rates in patients with COVID-19 without increasing the incidence of adverse events, thereby demonstrating a good overall safety profile (Wen et al., 2022). However, additional research is required to confirm these findings. Despite no longer being recommended, hydroxychloroquine and lopinavir-ritonavir were frequently utilized during the COVID-19 outbreak. According to the incidence data on liver damage summarized in this research, they have caused severe damage. It may aid in rationalizing the usage of repurposed medications when confronted with a new and severe epidemic.

The history of documented drug use is replete with drug-induced diseases caused by drug exposure and drug resistance owing to drug abuse, ending in the predicament of having no available medications. This rendered the drugs used to treat the sickness hazardous to the health of the patient instead. DILI is a prevalent drug-induced disease, and the authors suggest that it is crucial to use drugs with cautious discretion in dosage, to consider drug-drug interactions if combination, and to strike a balance between the therapeutic effects and toxicity of drugs in order to reduce the incidence of DILI. It is recommended that patients are also closely monitored, with the aim of early detection and treatment to minimize the risk of DILI.

This review summarizes the incidence of liver function abnormalities in COVID-19 patients caused by several antiviral drugs, including favipiravir, remdesivir, lopinavir/ritonavir, and hydroxychloroquine, while also providing thorough speculation of the underlying mechanism and suggesting reasonable clinical management. This advances the systematic understanding of DILI in COVID-19 patients and directs the clinical care of medical practitioners. There are also some limitations, such as the fact that this study’s data were not subjected to a systematic analysis because there were insufficient and inconsistently high-quality clinical trials evaluating adverse reactions; additionally, the mechanism hypothesis is largely based on the results of *in vitro* experiments and needs to be confirmed by additional clinical studies.
